# Circulating Mesenchymal Stem Cells Microparticles in Patients with Cerebrovascular Disease

**DOI:** 10.1371/journal.pone.0037036

**Published:** 2012-05-15

**Authors:** Suk Jae Kim, Gyeong Joon Moon, Yeon Hee Cho, Ho Young Kang, Na Kyum Hyung, Donghee Kim, Ji Hyun Lee, Ji Yoon Nam, Oh Young Bang

**Affiliations:** 1 Department of Neurology, Samsung Medical Center, Sungkyunkwan University School of Medicine, Seoul, South Korea; 2 Clinical Research Center, Samsung Biomedical Research Institute, Seoul, South Korea; 3 Clinical Trial Center, Samsung Medical Center, Seoul, South Korea; 4 Samsung Advanced Institute for Health Sciences and Technology, Seoul, South Korea; University of Medicine and Dentistry of New Jersey, United States of America

## Abstract

Preclinical and clinical studies have shown that the application of CD105^+^ mesenchymal stem cells (MSCs) is feasible and may lead to recovery after stroke. In addition, circulating microparticles are reportedly functional in various disease conditions. We tested the levels of circulating CD105^+^ microparticles in patients with acute ischemic stroke. The expression of CD105 (a surface marker of MSCs) and CXCR4 (a CXC chemokine receptor for MSC homing) on circulating microparticles was evaluated by flow cytometry of samples from 111 patients and 50 healthy subjects. The percentage of apoptotic CD105 microparticles was determined based on annexin V (AV) expression. The relationship between serum levels of CD105^+^/AV^−^ microparticles, stromal cells derived factor-1α (SDF-1α), and the extensiveness of cerebral infarcts was also evaluated. CD105^+^/AV^−^ microparticles were higher in stroke patients than control subjects. Correlation analysis showed that the levels of CD105^+^/AV^−^ microparticles increased as the baseline stroke severity increased. Multivariate testing showed that the initial severity of stroke was independently associated with circulating CD105^+^/AV^−^ microparticles (OR, 1.103 for 1 point increase in the NIHSS score on admission; 95% CI, 1.032–1.178) after adjusting for other variables. The levels of CD105^+^/CXCR4^+^/AV^−^ microparticles were also increased in patients with severe disability (r = 0.192, p = 0.046 for NIHSS score on admission), but were decreased with time after stroke onset (r = −0.204, p = 0.036). Risk factor profiles were not associated with the levels of circulating microparticles or SDF-1α. In conclusion, our data showed that stroke triggers the mobilization of MSC-derived microparticles, especially in patients with extensive ischemic stroke.

## Introduction

Adult stem cells circulate between organs for repair and maintenance of tissues [1], [2]. Circulating progenitor/stem cells have been reported in patients with ischemic stroke [3]–[8] and myocardial infarction [9]–[11]. Human acute stroke is followed by large and bursting mobilization of peripheral blood immature hematopoietic CD34^+^ cells, colony-forming cells and long-term culture initiating cells [6], and the extent of such mobilization is directly related to neurological and functional recoveries [7], [8]. Additionally, it has been reported that there is a correlation between stroke severity and the number of circulating cells expressing early stem cell markers [3].

Mesenchymal stem cells (MSCs) may also circulate in patients with acute ischemic stroke. The circulating MSCs population was reportedly higher in patients with various disease conditions [12]–[14]. Furthermore, the neurorestorative capacity of MSCs, such as angiogenesis, axonal regeneration, and trophic factor expression, was increased in MSCs from stroke rats when compared with normal rats [15]. Preclinical studies showed that increasing circulating CD105^+^ (endoglin, a surface marker of MSCs) MSCs by exogenous administration was effective in recovery after stroke. Moreover, intravenous application of CD105^+^ MSCs induced neurogenesis and angiogenesis in animal models of stroke [1], [16]. We also recently reported that transplantation of autologous MSCs was feasible and may be effective in stroke patients with severe neurologic deficits [17], [18]. However, although circulating MSCs have been reported to be dramatically upregulated by hypoxia in rats [19], few studies have been conducted to investigate circulating MSCs in stroke patients.

It was previously believed that microparticles were artifacts formed by apoptotic cell death. However, this view changed when the release of these small vesicles was recognized to result from specific process [20], [21]. Microparticles may be a window for target cells/organs. For example, acute myelogenous leukemia patients contain elevated blood and bone marrow plasma levels of CXCR4^+^ microparticles. Additionally, a relationship between CXCR4 expression on parent cells and CXCR4^+^ microparticles was observed [22]. Circulating microparticles are also known to be functional in various diseases [21]–[24]. However, circulating CD105^+^ or CXCR4^+^ (a CXC chemokine receptor for MSC homing) microparticles have not been investigated in vivo in patients with cerebrovascular disease.

In this study, we tested the hypothesis that circulating CD105^+^ and CXCR4^+^ microparticles are upregulated in acute stroke patients. The percentage of apoptotic microparticles was also assessed because it has been reported that apoptotic circulating progenitor/stem (annexin V^+^/CD34^+^) cells are increased after acute myocardial infarcts [25], and apoptotic microparticles may reflect target cell dysfunction (e.g. annexin V^+^ endothelial microparticles in stroke patients with endothelial dysfunction) [26]. In the present study, the relationship between serum levels of CD105^+^/annexin V (AV)^−^ microparticles, stromal cells derived factor-1α (SDF-1α, CXCL 12, a chemoattractant of MSCs), and the extensiveness of cerebral infarcts was evaluated.

## Methods

### Study Population

Subjects were recruited from the patient population of a university hospital. We enrolled 111 patients (46 women, mean age 66.0±11.3 years) afflicted with ischemic cerebrovascular events who were admitted within 24 hours of the onset of the first symptom. A group of 50 healthy subjects served as a control. Stroke risk factors and National Institute of Health Stroke Scale (NIHSS) score were evaluated and peripheral blood samples were collected from all participants at the time of admission (mean ± SD, 3.21±2.28 days after stroke onset). All human samples were used in accordance with procedures approved by the Institutional Review Boards in Samsung Medical Center.

All the patients with ischemic cerebrovascular events underwent electrocardiography, echocardiography and brain MRI (3.0-tesla, Achieva, Philips Medical Systems), including diffusion-weighted imaging (DWI) and MR angiography of the cervical and intracranial vessels. MRI volume measurements were conducted by one of the authors who was blinded to the clinical information using a computer-assisted volumetric analysis program (Medical Image Processing, Analysis and Visualization, version 2.1, CIT, NIH). Each patient gave written, informed consent for his or her involvement and the Institutional Review Boards in Samsung Medical Center approved the study.

### Flow Cytometry

The expression of CD105, CXCR4 and AV on circulating microparticles was evaluated by flow cytometry. To preclude the possibility that CD105^+^ microparticles are of cerebral endothelial cell-origin [27], [28], we tested the correlation between CD105^+^ microparticles and endothelial microparticles (CD 31^+^/CD42b^−^). Double labeling with anti-CD42b was conducted to exclude CD31^+^ microparticles of platelet origin. Platelet-derived microparticles were assessed with anti-CD62P. Confirmation of the cellular origin of CD105^+^ microparticles was made by the presence of positive markers (CD90 and CD73), and the absence of negative markers (VE-cadherin (CD144) and KDR) for MSCs.

Citrated whole blood was collected and centrifuged at 1800 g for 15 minutes to prepare platelet-poor plasma [26]. Plasma samples of 250 µl were thawed and centrifuged for 10 min at 19,800 g and 10°C to collect the microparticles [27]. The microparticle pellet was resuspened with 20 µl of phosphate buffered saline (PBS). Microparticles (5 µl) were then incubated with fluorescent monoclonal antibodies (5 µl each): phycoerythrin (PE)-labeled anti-CD31 (555446; BD Biosciences, San Jose, CA), fluorescein isothiocyanate (FITC)–labeled anti-CD42b (555472; BD Biosciences), allophycocyanin (APC)-labeled anti-CD90 (559869; BD Biosciences), APC-labeled AV (550475; BD Biosciences), PE-labeled anti-CD62P (P-selectin; 55524; BD Biosciences), PE-labeled anti-CD184 (CXCR4; 555974; BD Biosciences), APC-labeled anti-CD90 (559869; BD Biosciences), PE-labeled anti-CD144 (VE-cadherin; 560410; BD Biosciences), APC-labeled anto-CD73 (560847; BD Biosciences), PE-labeled KDR (FAB357P, R&D Systems, Minneapolis, MN), and FITC-labeled anti-CD105 (Endoglin; MCA1557F; Serotec, Oxford, UK).

**Figure 1:**
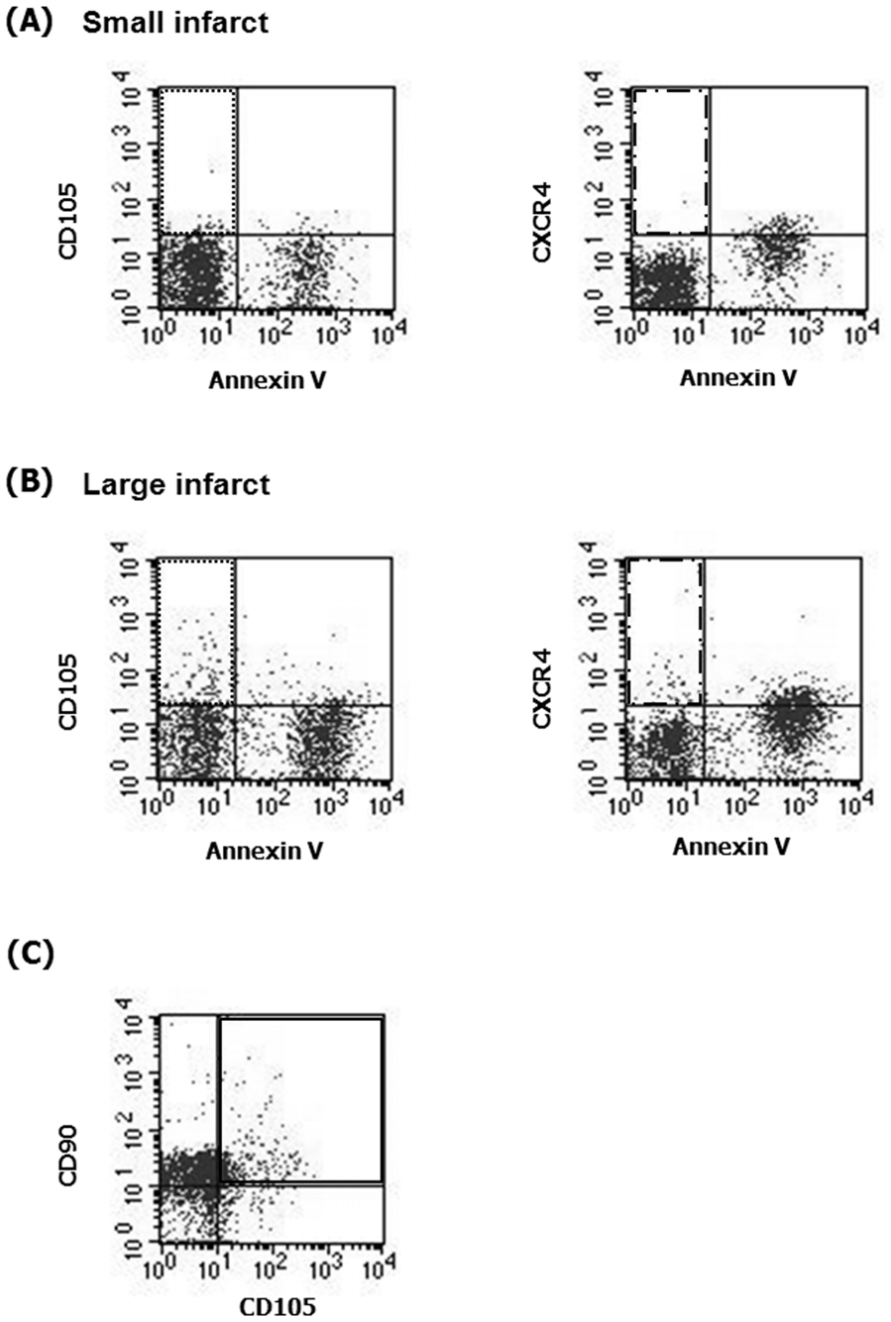
Typical examples of flow cytometric analysis. The levels of circulating CD105^+^/AV^−^ (Dotted rectangle) and CXCR4^+^/AV^−^ (Dashed and dotted rectangle) were greater in patients with (B) large infarct than in those with (A) small infarct. (C) Most CD105^+^ microparticles were CD90^+^ (Rectangle). See text for abbreviations.

The samples were incubated in the dark for 15 min at room temperature, after which 500 µl 1× binding buffer was added, and the samples were analyzed on a FACS Calibur flow cytometer using the CellQuest software (BD Biosciences). Microparticles were analyzed using a protocol with both forward scatter (FSC) and side scatter (SSC) in logarithmic mode. Standard beads 1.0 µm in diameter (Sigma; Molecular Probes, Eugene, OR, USA) were used for estimation of the microparticle size. For each sample, 10,000 events were acquired. Microparticle levels were corrected for an isotype control antibody, and dot plots with control antibody. Microparticles smaller than 1 µm were quantified in specific populations (CD105^+^, CXCR4^+^, CD105^+^/CXCR4^+^, CD105^+^/90^+^, CD105^+^/144^−^, CD105^+^/144^−^/90^+^, CD105^+^/KDR^−^, CD105^+^/73^+^, CD105^+^KDR^−^/73^+^, CD31^+^/CD42b^−^ and CD62P^+^). Based on the number of events (N) in the upper right (marker-positive and AV-positive) quadrant of the flow cytometric analysis (FL-2 vs. FL-4, corrected for isotype control antibody binding and autofluorescence), the number of microparticles per liter of plasma was calculated as: n/L = N×(20/5)×(550/V)×(10^6^/250), where 5 (µL) is the volume of microparticle suspension, 20 is the total volume of washed microparticle suspension, 550 is the total volume in the tube before analysis, V is the sample volume analyzed, 10^6^ is the number of microliters per liter, and 250 is the original volume of plasma (Figure 1) [29], [30]. Laboratory personnel who conducted the blood assays were unaware of the subject's clinical or laboratory data.

### Plasma Concentrations of SDF-1α

SDF-1α–CXCR4 axis was associated with MSC homing to the infracted hemisphere [31]. Circulating CXCR4^+^ stem cells were chemoattracted from peripheral blood to the brain by SDF-1α, which is highly expressed in injured brain areas.

The plasma levels of the SDF-1α were evaluated to determine if they were associated with the level of circulating CD105^+^/CXCR4^+^ microparticles. The concentrations of SDF-1α were measured using a commercially available, human SDF-1α Quantikine immunoassays ELISA kit (R&D Systems, Minneapolis, MN) according to the manufacturer's protocols.

### In vitro assay for CD105^+^ microparticles derived from human MSCs

#### A. Preparation of ischemic brain extract

Ischemic brain tissue extracts were obtained 3 days after 90 min transient middle cerebral artery occlusion. The ipsilateral hemispheres were homogenized together by adding Dulbecco's modified Eagle's medium (DMEM) (150 mg/mL) on the ice. After centrifugation for 10 min at 10,000 g at 4°C, the supernatants were collected and stored at −70°C [32], [33].

#### B. Cell culture and ischemic conditioning

The same methods were used in a previous work [17] for human bone marrow MSCs (hMSCs) cell culture. Briefly, hMSCs were cultured in a 175 cm^2^ flask (Falcon, Franklin Lakes, NJ) with low-glucose DMEM (Gibco, New York, NY) containing 10% fetal bovine serum (Gibco, New York, NY) and 1% penicillin-streptomycin (Sigma, St Louis, MO) in a humidified incubator at 37°C under 5% CO2. Nonadherent cells were removed when medium was exchanged at the 5th day. When the primary hMSCs expanded to 80% confluence, they were harvested and subcultured.

hMSCs were incubated with Knockout DMEM (serum free media, Gibco, New York, NY), 10% or 20% ischemic brain extracts (BE). After 24 h incubation, conditioned media were cleared from cell fragments by centrifugation at 2,500 g for 10 min at 10°C. The supernatants were stored at −70°C until future analysis.

### Statistical Methods

All data are presented as the mean ± SD or number (percentage) unless otherwise stated. The Shapiro-Wilk test was used to test for normal distribution of continuous variables. Because the distribution of microparticle levels was not normal (p<0.05), we included log values that showed a normal distribution. Correlations between two continuous variables were conducted using Pearson correlation coefficients. For each microparticle variable, we divided the levels into increasing quartiles. Comparisons between groups were analyzed by a Student's t-test or analysis of variance (ANOVA), and categorical variables were compared using the Pearson Chi-square test or Fisher's exact test. Analysis of covariance (ANCOVA) was used to compare age- and sex-adjusted mean values for microparticles between stroke patients and healthy subjects. Post hoc comparisons were also performed to test for differences between means among groups. A multivariable logistic regression model was used to evaluate the independent association between the initial severity of stroke and the levels of circulating CD105^+^/AV^−^ and CD105^+^/CXCR4^+^/AV^−^ microparticles. The potential confounding factors considered for inclusion in the model were risk factor profiles, time interval between the onset of stroke and sampling, and serum levels of SDF-1α. These factors were entered into a stepwise logistic regression model, and the factors that were not significant (p>0.2) were sequentially deleted from the full model. The results are given as odds ratio (OR), as estimates of relative risk with 95% CI. Significance was established at the p<0.05 level. All statistical analyses were conducted using the SPSS software (SPSS 13.0; SPSS, Chicago, IL).

## Results

### Characteristics of Study Population

The baseline characteristics and results of flow cytometric analysis are summarized in Table 1. The age was younger and female gender was more prevalent in the healthy subjects than in the stroke patients. However, the risk factor profiles and serum levels of SDF-1α did not differ between stroke patients and healthy subjects. In the gated particles, the level of CD105^+^/AV^−^ microparticles was significantly increased in stroke patients when compared to healthy subjects (p = 0.036) (Table 1).

**Table 1:** Study population characteristics.

	Acute stroke (n = 111)	Healthy subjects (n = 50)	*P*
Mean age ± SD, year	66.0±11.3	60.1±8.5	0.001
Female gender, n (%)	46 (41.4)	31 (62.0)	0.016
Hypertension, n (%)	67 (60.4)	24 (48.0)	0.143
Diabetes mellitus, n (%)	34 (30.6)	11 (22.0)	0.259
Dyslipidemia, n (%)	14 (12.6)	6 (12.0)	0.913
Atrial fibrillation, n (%)	13 (11.7)	2 (4.0)	0.098
SDF-1α, pg/ml	1678.3±483.5	1687.3±477.9	0.913
Microparticles, mean ± SD, per µl*			
CD105^+^	1.621±0.286	1.574±0.230	0.282
CD105^+^/AV^−^	1.232±0.278	1.118±0.214	0.036
CD105^+^/AV^+^	1.332±0.376	1.328±0.330	0.762
CD105^+^/CXCR4^+^	1.063±0.444	1.162±0.300	0.139
CD105^+^/CXCR4^+^/AV^−^	0.633±0.430	0.714±0.273	0.177
CD105^+^/CXCR4^+^/AV^+^	0.796±0.510	0.922±0.413	0.174

* Values after common logarithmic transformation.

Analysis of covariance was used to compare age- and sex-adjusted mean values for microparticles between groups.

See text for abbreviations.

### Microparticle Assay in Acute Stroke

**Figure 2:**
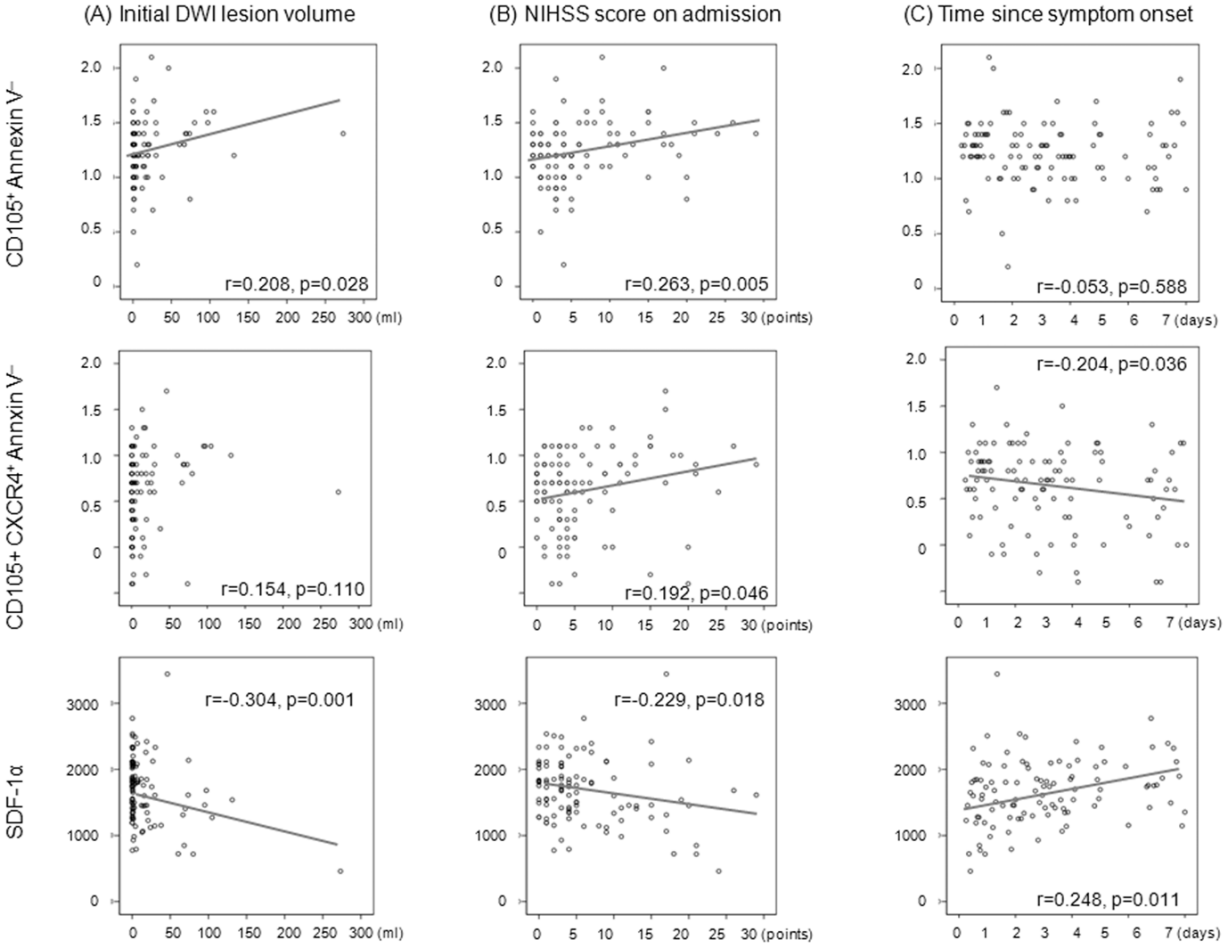
Correlation of microparticles with baseline stroke severity ((A) initial DWI lesion volume and (B) NIHSS score) and (C) time since stroke onset. * Microparticle values after common logarithmic transformation. See text for abbreviations.

We next searched for factors associated with a robust increase in CD105^+^/AV^−^ microparticles in acute stroke setting. Among patients with acute ischemic cerebrovascular disease, the levels of CD105^+^/AV^−^ microparticles were increased as the baseline stroke severity increased (r = 0.208, p = 0.028 for initial DWI lesion volume; r = 0.263, p = 0.005 for initial NIHSS score) (Figure 2). The levels of CD105^+^/CXCR4^+^/AV^−^ microparticles were also higher in patients with severe disability (r = 0.192, p = 0.046 for initial NIHSS score) (Figure 2). In addition, the levels of CD90+/AV^−^ and CD105+/90+/AV^−^ microparticles were positively correlated with initial DWI lesion volume (r = 0.349, p = 0.013; r = 0.415, p = 0.003, respectively).

**Figure 3:**
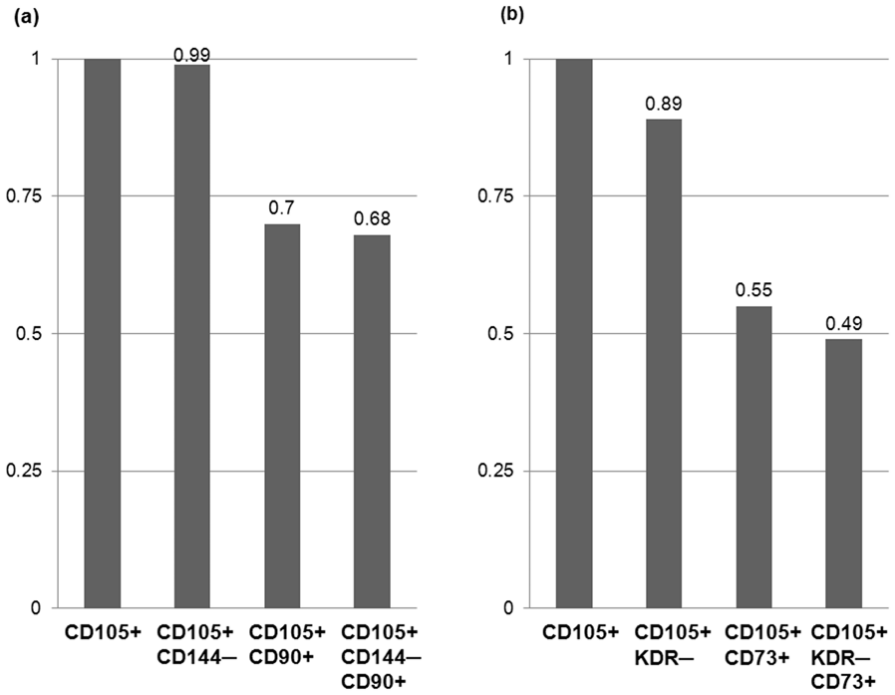
Cellular origin of CD105^+^ microparticles. To know the cellular origin of CD105^+^ microparticles, double labeling with anti-CD90, anti-CD73 (positive markers for MSCs), anti-CD144, or KDR (negative markers for MSCs), and triple labeling with anti-CD90 and anti-CD 144, or anti-CD73 and KDR were performed in patients with ischemic cerebrovascular disease. (a) Proportion of CD105^+^/144^−^, CD105^+^/90^+^, and CD105^+^/144^−^/90^+^ among CD105^+^ microparticles. (b) Proportion of CD105^+^/KDR^−^, CD105^+^/73^+^, and CD105^+^/KDR^−^/73^+^ among CD105^+^ microparticles. See text for abbreviations.

Bivariate correlation analysis of data from acute stroke patients showed that the CD105^+^ microparticle phenotypes were positively correlated (data not shown). However, there was no correlation between the level of CD31^+^/CD42^−^ (endothelial) microparticles and CD105^+^/AV^−^ (r = −0.031, p = 0.764) or CD105^+^/CXCR4^+^/AV^−^ (r = 0.044, p = 0.668) microparticles levels, suggesting that CD105^+^ microparticles originated from bone marrow rather than endothelial cells. Similarly, there was no correlation between the levels of CD62P^+^ (platelet) microparticles and CD105^+^ microparticle phenotypes. Moreover, most CD105^+^ microparticles were CD144^−^, KDR^−^, CD90^+^, and CD144^−^/CD90^+^ and approximately half of CD105+ microparticles were CD73^+^ and KDR^−^/73^+^ (Figure 1C and Figure 3).

### Multivariate Testing

Patients with ischemic cerebrovascular disease were stratified into four groups according to interquartile cutoff points of the distribution of CD105^+^/AV^−^ microparticle levels (Table 2). The levels of CD105^+^ microparticles increased as the baseline stroke severity increased (p<0.05 for both initial DWI lesion volume and initial NIHSS score). The level of SDF-1α and time between symptom onset and sampling did not differ among groups. The risk factor profile did not affect the levels of circulating microparticles (Table S1). A similar pattern was observed for CD105^+^/CXCR4^+^/AV^−^ microparticles (data not shown).

**Table 2:** Baseline characteristics determined by quartiles of log-transformed CD105^+^/AV^−^ microparticle levels in 111 patients with ischemic cerebrovascular disease.

CD105^+^/AV^−^, per µl*	Q1 (<11.8)	Q2 (11.9–17.3)	Q3 (17.5–25.1)	Q4 (>25.1)	*P*
No. of patients	28	36	21	26	
Age	64.8±10.2	67.4±9.8	65.4±11.5	66.3±13.9	.847
Female gender, n (%)	14 (50)	10 (35.7)	11 (40.7)	11 (39.3)	.733
Risk factor profile, n (%)					
Hypertension	18 (64.3)	19 (67.9)	13 (48.1)	17 (60.7)	.471
Diabetes	8 (28.6)	12 (42.9)	7 (25.9)	7 (25.0)	.436
Dyslipidemia	4 (14.3)	5 (17.9)	4 (14.8)	1 (3.6)	.371
Atrial fibrillation	0 (0)	2 (7.1)	6 (22.2)	5 (17.9)	.023†
Baseline stroke severity					
DWI lesion volume, ml	7.7±15.8	9.5±25.1	12.9±24.0	33.81±57.5	.020
NIHSS score	4.71±5.11	4.25±4.20	6.00±7.29	9.64±7.51	.006
Time since symptom onset, days	3.73±2.21	3.17±2.03	2.47±2.20	3.38±2.58	.239
SDF-1α, pg/ml	1754.9±432.2	1704.8±384.0	1574.8±441.4	1677.7±636.9	.594
Endothelial microparticles, per µl*					
CD31^+^/CD42^−^	2.30±0.50	2.32±0.42	2.37±0.24	2.15±0.34	.283
CD31^+^/CD42^−^/AV^−^	0.67±0.73	0.57±0.12	0.45±0.10	0.31±0.60	.352
CD31^+^/CD42^−^/AV^+^	2.24±0.60	2.31±0.42	2.36±0.25	2.14±0.35	.176
Platelet microparticles, per µl*					
CD62P^+^	1.53±0.468	1.43±0.38	1.58±0.42	1.35±0.34	.167
CD62P^+^/AV^−^	0.38±0.51	0.17±0.49	0.24±0.39	0.28±0.32	.366
CD62P^+^/AV^+^	1.48±0.48	1.38±0.41	1.55±0.43	1.30±0.36	.148

* Values after common logarithmic transformation of the number of microparticles (per µl).

† related to larger infarct size in patients with atrial fibrillation.

Multivariate testing showed that the initial severity of stroke was independently associated with the level of circulating CD105^+^/AV^−^ microparticles (OR, 1.103 for 1 point increase in the NIHSS score on admission; 95% CI, 1.032–1.178) after adjusting for other variables, including the risk factor profiles, time after the onset of stroke, and serum levels of SDF-1α (Table 3). Other factors did not significantly add value to the level of circulating CD105^+^/AV^−^ microparticles.

**Table 3:** Multivariate testing: odds ratio (95% CI) for the highest quartile of CD105^+^ microparticle levels.

	CD105^+^/AV^−^ microparticles			CD105^+^/CXCR4^+^/AV^−^ microparticles		
	Crude	Multivariate	*P*	Crude	Multivariate	*P*
Initial DWI lesion volume, ml	1.008 (0.990–1.027)			0.999 (0.983–1.015)		
NIHSS score on admission	1.083 (0.981–1.196)	1.103 (1.032–1.178)	.*004*	1.097 (0.996–1.208)	1.086 (1.018–1.159)	*0.015*
Atrial fibrillation	2.227 (0.527–9.420)			3.819 (0.961–15.17)		
SDF-1α, pg/ml	1.000 (0.999–1.001)			1.000 (0.999–1.001)		
Time since symptom onset, days	1.208 (0.963–1.517)			1.151 (0.914–1.448)		

See text for abbreviations.

### CXCR4^+^microparticles and SDF-1α

The levels of CD105^+^/CXCR4^+^ microparticles did not differ between the stroke patients and healthy subjects (Table 1). However, the proportion of CD105^+^/CXCR4^+^ microparticles among CXCR4^+^ microparticles was higher in the acute stroke patients than in healthy subjects (29.5±17.9% vs. 21.0±16.9%, p = 0.011).

In the acute stroke setting, there was a poor correlation between the serum level of CD105^+^/CXCR4^+^/AV^−^ microparticles and SDF-1α (r = −0.042, p = 0.674). The level of SDF-1α increased with time after stroke onset (r = 0.248, p = 0.011), whereas the levels of CD105^+^/CXCR4^+^/AV^−^ microparticles decreased with time after stroke onset (r = −0.204, p = 0.036) (Figure 2).

### Ischemic brain tissue extracts induce in vitro CD105^+^ microparticles derived from hMSCs

**Figure 4:**
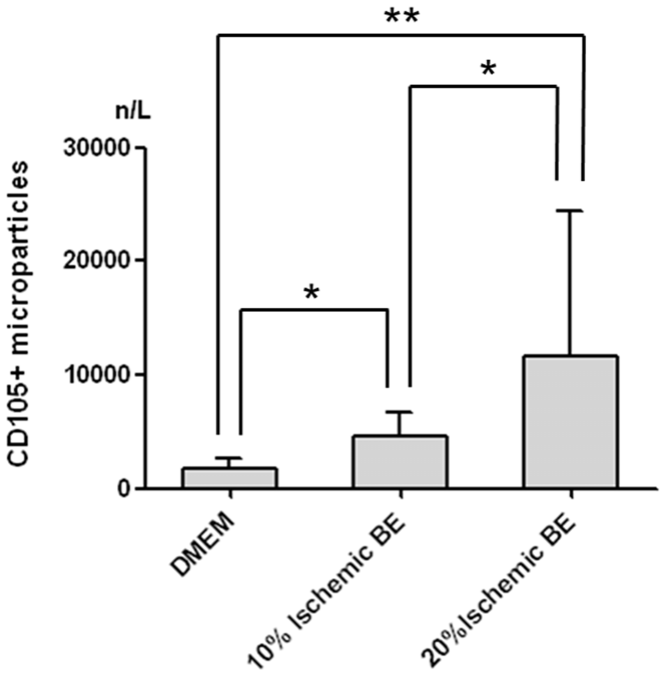
In vitro assay for CD105^+^ microparticles derived from hMSCs. Compared to hMSCs treated by Knockout DMEM, those conditioned by ischemic BE released more CD105^+^ microparticles. Values are median and interquartile range; n = 6 per group. *p<0.01, **p<0.001. See text for abbreviations.

In order to further confirm that CD105^+^ microparticles of hMSCs are increased by ischemic injury, in vitro assay was conducted. Compared to hMSCs treated by Knockout DMEM, those conditioned by ischemic BE released more CD105^+^ microparticles (p<0.01, DMEM vs. 10% ischemic BE; p<0.001, DMEM vs. 20% ischemic BE). Moreover, hMSCs with 20% ischemic BE derived more CD105^+^ microparticles than those with 10% ischemic BE (p<0.01) (Figure 4).

## Discussion

The main finding of this study was that there was a significant relationship between circulating CD105^+^/AV^−^ microparticles and the extent of infarct. The increase in the level of circulating CD105^+^ microparticles after acute stroke may be caused by (a) increased circulating stem cells as a self-repair response after stroke or by (b) increased apoptosis of circulating stem cells in response to toxic conditions. Atheroprotective endothelial progenitor cells (EPCs) may undergo fragmentation into microparticles when exposed to cardiovascular risk factors [34]. Moreover, increased microparticle shedding from EPCs may reduce circulating EPCs levels and impair vascular repair processes. In patients with extensive myocardial infarction, apoptotic progenitor cells (CD34^+^/AV^+^) were increased, suggesting that progenitor CD34^+^ cells are mobilized from the bone marrow but destroyed by apoptosis due to increased oxidative stress [25].

Recently, the understanding of the role of microparticles in various diseases has increased. Indeed, there is increasing evidence that microparticles are functional [22] and facilitate intercellular communication [35]. Platelet-derived and endothelial-derived microparticles as well as microparticles from ischemic muscle have also been found to promote vasculogenesis [24], [36], [37]. Interestingly, however, the results of the present study showed that circulating CD105^+^/AV^−^ (not apoptotic) microparticles were increased in patients with extensive stroke, but that CD105^+^/AV^+^ microparticles were not associated with the extent of infarct. There have been growing evidences that AV+ and AV− microparticles are produced under different circumstances and have distinct properties or functions [38], [39]. Since CD105+/AV− microparticles were associated with the extent of stroke, but not CD105+/AV+, we simply assume that extensive stroke may trigger the formation of more AV− microparticles from MSCs. However, the precise mechanism should be elucidated through future studies.

Although circulating EPCs has been reported in patients with ischemic insults, no studies have investigated circulating CD105^+^ or CXCR4^+^ cells or microparticles in stroke patients to date. Our finding of increased circulating CD105^+^ microparticle levels in patients following severe stroke may reflect the increased level of circulating MSCs or MSC-origin microparticles. Elevated CD105^+^ microparticles may be derived from either bone marrow or cerebral endothelial cells [27], [28]. However, our results showed that CD105^+^ microparticles were also positive for CD90 or CD73 (positive markers for MSCs) and negative for CD144 or KDR (negative markers for MSCs). In addition, there was no correlation between endothelial/platelet microparticles and CD105^+^ microparticle phenotypes. Moreover, *in vitro* assay showed that CD105^+^ microparticles derived from hMSCs were increased by ischemic BE with dose-dependent manner. Together with the fact that most bone marrow MSCs are CD105^+^, it is conceivable that CD105^+^ microparticles originated from bone marrow MSCs rather than endothelial cells. To the best of our knowledge, this is the first report of clinical evidence that MSCs are mobilized into peripheral blood in patients after stroke.

It is well known that the SDF-1α receptor, CXCR4, is expressed in bone marrow stromal cells. These cells migrated into ischemic areas where chemokine SDF-1α expression has increased, expressed neuronal or glial markers, increased neurogenesis and reduced scar thickness [40]. Our finding of a poor correlation between SDF-1α and CD105^+^/CXCR4^+^ microparticles was unexpected and may have been caused by several reasons. First, a different peak time between SDF-1α and CD105^+^/CXCR4^+^/AV^−^ microparticles after stroke may prevent a putative relationship between serum chemokine and its receptor on microparticles. In the present study, the SDF-1α levels increased, but the CD105^+^/CXCR4^+^/AV^−^ microparticles decreased with time after the onset of stroke. Second, our data showed that approximately 70% of CD105^+^ microparticles did not express CXCR4. In addition to the difference in peak time between SDF-1α levels and CD105^+^/CXCR4^+^/AV^−^ microparticles, there may be chemokines for CD105^+^ microparticles other than the SDF-1α/CXCR4 axis. Our *in vitro* study suggests the possible existence of chemokines other than SDF-1α that mediated the circulating levels of CD105^+^/AV^−^ microparticles. *In vitro* migration analysis using Transwell® comparing the chemoattractive capacity between ischemic brain extract and SDF-1α showed that the migration of MSCs was higher when ischemic brain extract was used than SDF-1α, and was not blocked by the treatment of AMD3100 (an SDF-1α antagonist) (Figure S1). These findings suggest that chemokines other than SDF-1α also mediated the migration of MSCs. However, further studies are needed to identify chemokines other than SDF-1α in stroke patients.

It should be noted that our study has several limitations. First, laboratory methods for the isolation and detection of microparticles have not been settled to date, specifically in terms of the centrifugation method and definition of microparticles. Most studies have defined microparticles based on their size (less than 1 µm) [25], [26], [34], [41], although some have defined them as AV^+^ microparticles [24]. It has been reported that circulating apoptotic (AV^+^) progenitor cells were increased in response to acute ischemic insult (acute coronary syndrome, especially in extensive coronary artery disease), suggesting that AV^+^ microparticles could be a marker for apoptotic or “non-functional” microparticles [25]. However, Leroyer et al. recently showed that microparticles generated locally after limb muscle ischemia (mostly from endothelial cells) triggered vasculogenesis, and they suggest that microparticles may interact with bone marrow cells through phosphatidyl serine (AV-binding site)-dependent binding [24]. Further studies are needed to compare the role of AV^+^ and AV^−^ microparticles in recovery after stroke. Second, although our data showed that circulating CD105^+^ microparticles were higher in patients with extensive lesions, microparticles could also be generated by systemic inflammatory response or ischemic brain injury. However, parameters indicative for infection (such as C-reactive protein) did not differ in response to different CD105^+^ microparticle levels, making a systemic cause of the observed differences unlikely. Finally, peripheral circulating CD105^+^ cells were not measured in this study.

In conclusion, our data showed that stroke triggers the mobilization of bone marrow-derived microparticles, and the level of CD105^+^/AV^−^ microparticles increased after extensive infarcts, but decreased with time. Further studies are needed to evaluate the role of bone marrow-origin microparticles in recovery after stroke.

## Supporting Information

Figure S1 **Migration assay using rat bone marrow mesenchymal stem cells (MSCs).** Migration of rat bone marrow MSCs was test using SDF-1α (*B and E*) and rat ischemic brain extracts (*C and F*) as chemokines. Compared to control group Knockout DMEM (*A and D*), migration of MSCs was increased in both SDF-1α and ischemic brain extracts group. The degree of increase in migration was greater in ischemic brain extracts group than in SDF-1α group. After treatment of SDF-1α antagonist (20 µM of AMD 3100), the migration of MSCs was nearly completely blocked in the SDF-1α group (*E*) but not in the ischemic brain extracts group (*F*) (see also *right panel*). These findings suggest that chemokines other than SDF-1α play a role in the migration of MSCs. Values were mean ± SEM; n = 4 per group; *p<0.01 vs. AMD3100 untreated control. **Methods** **for migration assay:** Migration assays were performed in transwell system (Corning Life Sciences, Acton, MA), the lower side of the transwell filter with 8 µm pore was coated for 1 hour at 37°C with 50 µg/ml Fibronectin (Sigma, St. Louis, MO). Rat bone marrow MSCs (5×10^4^ cells) were placed in the upper chamber, and 600 µl of migration medium with chemotactic factors or brain extract supernatant were placed in the bottom chamber. Migration observed in Knockout DMEM alone served as negative control. We evaluated the chemotactic activity of SDF-1α (150 ng/ml). To block chemotactic activity by receptor CXCR4, 20 µM of AMD3100 (Sigma, St. Louis, MO) was used. After 4 hours, assays were terminated by removal of the medium from the upper wells and filters were washed with PBS. Cells remaining on the upper face of the filters were removed with a cotton wool swab. Filters were fixed with methanol by submersion and stained with toluidine blue (Sigma, St. Louis, MO) solution for 5 minutes and then air dried. Filters cut out with a scalpel were mounted onto glass slides, putting the lower face on the top. Stained cells in 5 fields were counted manually under ×100 magnification using light microscopy. Data were expressed as percentages of cells related to that of the negative control.
(TIF)

Table S1 **Microparticle levels (mean±SD, per µl^a^) and risk factor profiles.**
(DOC)
