# Supplementary figures and images for: Circulating Mesenchymal Stem Cells Microparticles in Patients with Cerebrovascular Disease
Source: PLoS One. 2012 May 15;7(5):e37036. doi: 10.1371/journal.pone.0037036 (PMC3352849; doi:10.1371/journal.pone.0037036)

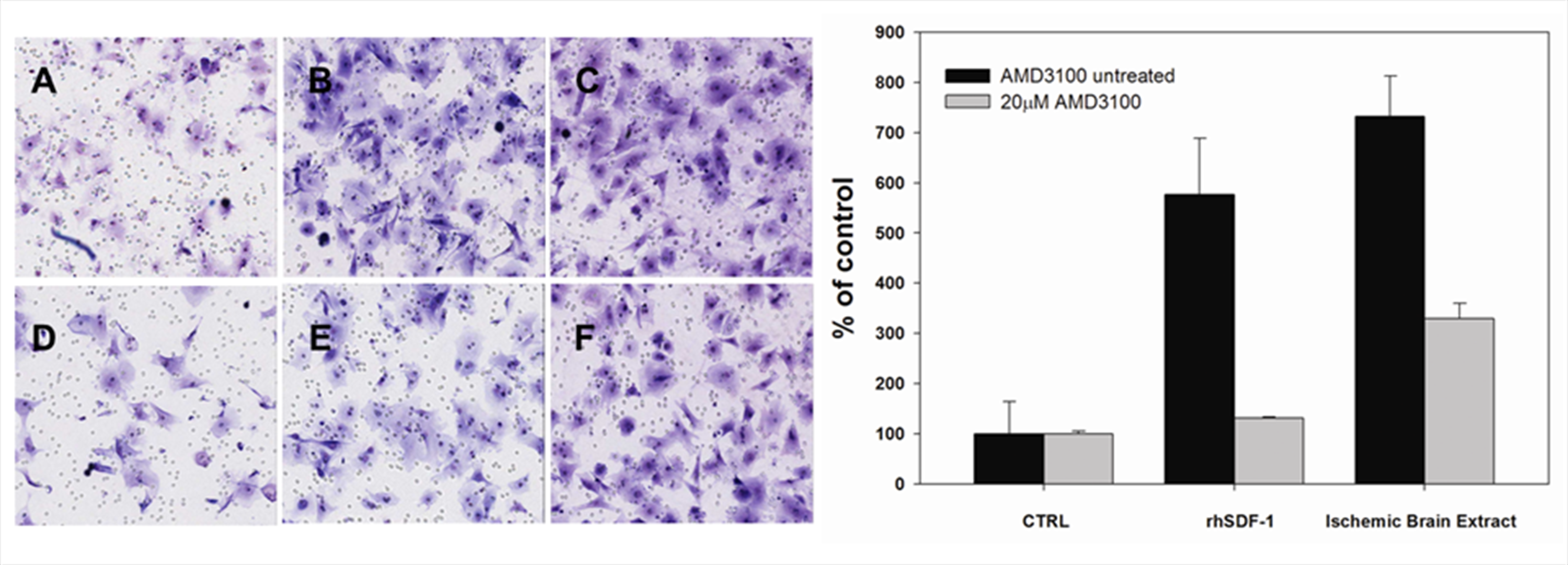

Supplement: Figure S1 — Migration assay using rat bone marrow mesenchymal stem cells (MSCs). Migration of rat bone marrow MSCs was test using SDF-1α (B and E) and rat ischemic brain extracts (C and F) as chemokines. Compared to control group Knockout DMEM (A and D), migration of MSCs was increased in both SDF-1α and ischemic brain extracts group. The degree of increase in migration was greater in ischemic brain extracts group than in SDF-1α group. After treatment of SDF-1α antagonist (20 µM of AMD 3100), the migration of MSCs was nearly completely blocked in the SDF-1α group (E) but not in the ischemic brain extracts group (F) (see also right panel). These findings suggest that chemokines other than SDF-1α play a role in the migration of MSCs. Values were mean ± SEM; n = 4 per group; *p<0.01 vs. AMD3100 untreated control. Methods for migration assay: Migration assays were performed in transwell system (Corning Life Sciences, Acton, MA), the lower side of the transwell filter with 8 µm pore was coated for 1 hour at 37°C with 50 µg/ml Fibronectin (Sigma, St. Louis, MO). Rat bone marrow MSCs (5×10^4^ cells) were placed in the upper chamber, and 600 µl of migration medium with chemotactic factors or brain extract supernatant were placed in the bottom chamber. Migration observed in Knockout DMEM alone served as negative control. We evaluated the chemotactic activity of SDF-1α (150 ng/ml). To block chemotactic activity by receptor CXCR4, 20 µM of AMD3100 (Sigma, St. Louis, MO) was used. After 4 hours, assays were terminated by removal of the medium from the upper wells and filters were washed with PBS. Cells remaining on the upper face of the filters were removed with a cotton wool swab. Filters were fixed with methanol by submersion and stained with toluidine blue (Sigma, St. Louis, MO) solution for 5 minutes and then air dried. Filters cut out with a scalpel were mounted onto glass slides, putting the lower face on the top. Stained cells in 5 fields were counted manually under ×100 magnification [file pone.0037036.s001.tif]
