# Supplementary material for: Circulating Mesenchymal Stem Cells Microparticles in Patients with Cerebrovascular Disease
Source: PLoS One. 2012 May 15;7(5):e37036. doi: 10.1371/journal.pone.0037036 (PMC3352849; doi:10.1371/journal.pone.0037036)
Supplement: Table S1 — Microparticle levels (mean±SD, per µla) and risk factor profiles. (DOC) [file pone.0037036.s002.doc]

**Supplementary Table S1. Microparticle levels (mean±SD, per μl^a^) and risk factor profiles**

|  | **Absence** | **Presence** | ***P*** |
| --- | --- | --- | --- |
| Hypertension, |  |  |  |
| CD105^+^ | 1.645 ± 0.259 | 1.604 ± 0.303 | 0.463 |
| CD105^+^/AV^-^ | 1.241 ± 0.255 | 1.227 ± 0.295 | 0.796 |
| CD105^+^/AV^+^ | 1.364 ± 0.350 | 1.310 ± 0.394 | 0.469 |
| CD105^+^/CXCR4^+^ | 1.093 ± 0.396 | 1.043 ± 0.475 | 0.565 |
| CD105^+^/CXCR4^+^/AV^-^ | 0.623 ± 0.435 | 0.640 ± 0.430 | 0.838 |
| CD105^+^/CXCR4^+^/AV^+^ | 0.832 ± 0.483 | 0.773 ± 0.529 | 0.555 |
| SDF-1α | 1662.0±542..9 | 1701.0±472.2 | 0.707 |
| Diabetes mellitus |  |  |  |
| CD105^+^ | 1.647 ± 0.305 | 1.562 ± 0.232 | 0.150 |
| CD105^+^/AV^-^ | 1.234 ± 0.299 | 1.229 ± 0.228 | 0.940 |
| CD105^+^/AV^+^ | 1.374 ± 0.386 | 1.235 ± 0.338 | 0.073 |
| CD105^+^/CXCR4^+^ | 1.119 ± 0.430 | 0.935 ± 0.454 | 0.043 |
| CD105^+^/CXCR4^+^/AV^-^ | 0.657 ± 0.437 | 0.579 ± 0.415 | 0.383 |
| CD105^+^/CXCR4^+^/AV^+^ | 0.869 ± 0.491 | 0.632± 0.522 | 0.024 |
| SDF-1α | 1719.8±502.4 | 1614.7±492.8 | 0.331 |
| Dyslipidemia |  |  |  |
| CD105^+^ | 1.641 ± 0.290 | 1.479 ± 0.216 | 0.046 |
| CD105^+^/AV^-^ | 1.242 ± 0.289 | 1.164 ± 0.182 | 0.329 |
| CD105^+^/AV^+^ | 1.352 ± 0.385 | 1.193 ± 0.287 | 0.141 |
| CD105^+^/CXCR4^+^ | 1.079 ± 0.440 | 0.950 ± 0.470 | 0.310 |
| CD105^+^/CXCR4^+^/AV^-^ | 0.631 ± 0.429 | 0.650 ± 0.455 | 0.875 |
| CD105^+^/CXCR4^+^/AV^+^ | 0.821 ± 0.511 | 0.629 ± 0.484 | 0.189 |
| SDF-1α | 1683.7±511.1 | 1695.9±437.3 | 0.933 |
| Atrial fibrillation, n (%) |  |  |  |
| CD105^+^ | 1.603 ± 0.283 | 1.754 ± 0.285 | 0.074 |
| CD105^+^/AV^-^ | 1.209 ± 0.278 | 1.408 ± 0.218 | 0.015 ^b^ |
| CD105^+^/AV^+^ | 1.314 ± 0.374 | 1.462 ± 0.380 | 0.186 |
| CD105^+^/CXCR4^+^ | 1.031 ± 0.449 | 1.308 ± 0.328 | 0.034 ^b^ |
| CD105^+^/CXCR4^+^/AV^-^ | 0.591 ± 0.427 | 0.946 ± 0.313 | 0.005 ^b^ |
| CD105^+^/CXCR4^+^/AV^+^ | 0.767 ± 0.521 | 1.015 ± 0.367 | 0.100 |
| SDF-1α | 1668.9±475.0 | 1804.4±660.6 | 0.381 |

^a^ Values after common logarithmic transformation.

^b^ related to large infarcts in atrial fibrillation-related stroke.
